# Explainable machine-learning predictions for complications after pediatric congenital heart surgery

**DOI:** 10.1038/s41598-021-96721-w

**Published:** 2021-08-26

**Authors:** Xian Zeng, Yaoqin Hu, Liqi Shu, Jianhua Li, Huilong Duan, Qiang Shu, Haomin Li

**Affiliations:** 1 The Children’s Hospital of Zhejiang University School of Medicine and National Clinical Research Center for Child Health, Hangzhou, China; 2 The College of Biomedical Engineering and Instrument Science, Zhejiang University, Hangzhou, China; 3 Department of Neurology, Rhode Island Hospital, Brown University, Providence, USA

**Keywords:** Cardiology, Risk factors

## Abstract

The quality of treatment and prognosis after pediatric congenital heart surgery remains unsatisfactory. A reliable prediction model for postoperative complications of congenital heart surgery patients is essential to enable prompt initiation of therapy and improve the quality of prognosis. Here, we develop an interpretable machine-learning-based model that integrates patient demographics, surgery-specific features and intraoperative blood pressure data for accurately predicting complications after pediatric congenital heart surgery. We used blood pressure variability and the k-means algorithm combined with a smoothed formulation of dynamic time wrapping to extract features from time-series data. In addition, SHAP framework was used to provide explanations of the prediction. Our model achieved the best performance both in binary and multi-label classification compared with other consensus-based risk models. In addition, this explainable model explains why a prediction was made to help improve the clinical understanding of complication risk and generate actionable knowledge in practice. The combination of model performance and interpretability is easy for clinicians to trust and provide insight into how they should respond before the condition worsens after pediatric congenital heart surgery.

## Introduction

Congenital heart disease is the most common form of major birth defect, affecting approximately 8 in 1000 live births worldwide^1^. Extraordinary advances in cardiovascular diagnostics and cardiothoracic surgery have increased the survival of newborns with congenital heart disease^2^. Nevertheless, the quality of treatment and prognosis after congenital heart surgery remains unsatisfactory, especially when complex surgery is performed^3,4^. Postoperative complications in congenital heart surgery have been inconsistently reported but have important contributions to mortality, hospital stay, cost and quality of life^5–7^. Heart centers with the best outcomes might not report fewer complications but rather have systems in place to recognize and correct complications before deleterious outcomes ensue^6^. In these cases, the early detection of deterioration after congenital heart surgery enables a prompt initiation of therapy, which may result in reduced impairment and earlier rehabilitation. Several scoring systems, such as the Risk Adjustment for Congenital Heart Surgery (RACHS-1) category^8^, the Aristotle Basic Complexity (ABC) score^9^, the European Association for Cardiothoracic Surgery and the Society of Thoracic Surgeons (STS-EACTS) mortality score^10^, and the STS-EACTS morbidity score^11^, have been developed and used to adjust the risk of in-hospital morbidity and mortality in the community. However, all these consensus-based risk models only focus on the procedure themselves and cannot be adjusted for specific patient characteristics such as lower weight^12^ and longer cardiopulmonary bypass (CPB)^13^, which were associated with worse outcomes after congenital heart surgery.

With the development of electronic health record (EHR) systems, abundant, complex, high-dimensional, and heterogeneous data are being captured during surgery and daily care. Researches using EHR data have shown that weight^12,14^, perioperative blood transfusions^15^, CPB^13,16^, and preoperative ejection fraction^17^ were associated with the risk of postoperative complications and mortality after congenital heart surgery. A machine learning-based predictive model^18^ has recently been used to identify independent risk factors and predict complications after congenital heart surgery. However, several gaps remain to be addressed. First, quantifying the effect of these risk factors in both a specific patient and a population in clinical practice is less explored. Second, highly intensive vital signs data during surgery were not fully utilized. Perioperative blood pressure control has been adopted as a significant clinical focus in congenital heart surgery. The previous model only used static features to predict postoperative complications and did not leverage intraoperative blood pressure data. Third, the previous model focused only on predicting whether patients had postoperative complications and did not address what kind of complications patients could experience. Clinicians might take different interventions for different complications, so it is of more clinical significance to predict the specific complications that patients will experience. Fourth, although machine learning model provides good prediction accuracy, its application in an actual clinical setting is limited because the prediction is difficult to interpret. Interpretable methods explain why a certain prediction was made for a patient, that is, a specific characteristic that led to the prediction.

**Figure 1:**
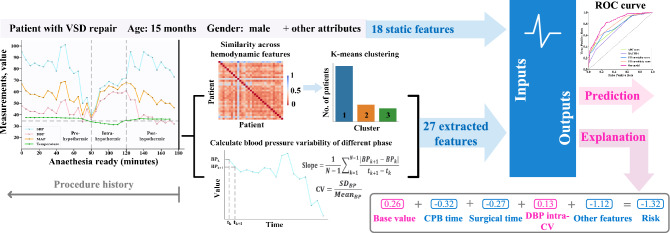
Schematic of data processing and workflow of our proposed model. We used soft-DTW to measure similarities of time-series hemodynamic data, and k-means clustering was applied to capture different dynamic patterns of hemodynamic data. Blood pressure variability was also used to measure blood pressure fluctuations within a period of surgery. All these extracted time-series features combined with the patient- and surgery-specific static features were used to build a predictive model of postoperative complications. An explanation was then built for each prediction. Pink features revealed that increased value is associated with an increased risk on the final prediction, whereas blue features decreased risk. The base value is the mean of the model output over the training dataset. The final prediction risk is the sum of the impacts of all features and base value, and then transformed into probability space; in this case, this patient has a 21% chance of having postoperative complications.

We aimed to develop and internally validate a machine learning model to predict the risk of complications and what kind of complications patients could experience using patient demographics, surgery-specific features, and intraoperative blood pressure data, all of which are routinely collected as part of medical records. In addition, to gain insight into the specific factors that contribute most to the model predictions, we used the feature attribution framework of SHAP (SHapley Additive exPlanations). The schematic of data processing and workflow of our proposed model was shown in Fig. 1. We believe the combination of model performance and interpretability is an important step forwards that enables the prediction of postoperative complications prediction to be more widely used in practice.

## Results

### Population characteristics

A total of 1964 patients with a median age of 11 months (IQR 4–26) were included in the final analyses, of which 582 (34.4%) patients developed postoperative complications, 134 (6.8%) patients developed cardiac complications, 131 (6.7%) patients developed rhythm complications, 432 (22.0%) patients developed lung complications, 90 (4.6%) patients developed infectious complications, and 155 patients developed other complications. Patient characteristics used in the final prediction model were shown in Table 1. The univariate analysis revealed that patients with postoperative complications were more likely to be boys, had lighter weight, shorter height, and younger age. Lower blood oxygen saturation levels before and after surgery were also associated with postoperative complications. Moreover, a longer surgical time, CPB time, and aortic cross-clamping time were associated with complications. The trajectory of blood pressure change during surgery and blood pressure variability of different phases were also associated with postoperative complications.

**Table 1:** Characteristics of cardiac patients stratified by postoperative complications (PC).

Characteristic	No PC (n = 1382)	PC (n = 582)	P value
Age (months)	14.4 [6.6, 36.2]	5.0 [2.2, 11.5]	< 0.001
Gender (male)	646 (47.2%)	312 (54.5%)	0.003
Height (cm)	77.0 [66.0, 95.0]	62.0 [55.0, 72.0]	< 0.001
Weight (kg)	9.5 [6.8, 13.8]	6.0 [4.1, 8.2]	< 0.001
**Main defect**			
ASD, secundum	651 (47.5%)	306 (53.5%)	0.204
VSD, type 2	579 (42.3%)	229 (40.0%)	1.908
PDA	240 (17.5%)	249 (43.5%)	< 0.001
PFO	343 (25.0%)	144 (25.2%)	5.676
VSD, type 1	217 (15.8%)	82 (14.3%)	2.406
TOF	32 (2.3%)	50 (8.7%)	< 0.001
**Main operation**			
VSD repair, patch	629 (45.9%)	289 (50.5%)	1.030
ASD repair, patch	414 (30.2%)	90 (15.7%)	< 0.001
PDA closure	242 (17.7%)	246 (43.0%)	< 0.001
PFO, primary closure	340 (24.8%)	138 (24.1%)	7.170
ASD repair, primary closure	242 (17.7%)	205 (35.8%)	< 0.001
VSD repair, primary closure	180 (13.1%)	25 (4.4%)	< 0.001
Valvuloplasty, mitral	53 (3.9%)	49 (8.6%)	< 0.001
Valvuloplasty, tricuspid	65 (4.7%)	36 (6.3%)	2.130
TAPVC repair	20 (1.5%)	43 (7.5%)	< 0.001
TOF repair, ventriculotomy, transannular patch	20 (1.5%)	40 (7.0%)	< 0.001
ABC score	6.0 [3.0,6.0]	6.0 [6.0,9.0]	< 0.001
**RACHS-1**			
1	400 (29.2%)	43 (7.5%)	< 0.001
2	824 (60.1%)	348 (60.8%)	4.920
3	137 (10.0%)	137 (24.0%)	< 0.001
4	9 (0.7%)	43 (7.5%)	< 0.001
5	0 (0.0%)	1 (0.2%)	1.480
STS-EACTS mortality score	0.2 [0.2, 0.4]	0.4 [0.2, 0.7]	< 0.001
STS-EACTS morbidity score	0.6 [0.2, 1.0]	1.1 [0.7, 1.3]	< 0.001
Surgical time (minutes)	120.0 [106.0,140.0]	152.5 [123.0,200.8]	< 0.001
CPB time (minutes)	56.0 [46.0, 70.0]	84.5 [62.0,127.8]	< 0.001
Aortic cross-clamping time (minutes)	36.0 [26.0, 47.0]	55.0 [41.0, 86.0]	< 0.001
Previous surgery	14 (1.0%)	10 (1.7%)	0.234
Preoperative LOS	4.0 [2.0, 6.0]	6.0 [4.0, 11.0]	< 0.001
**Surgical access route**			< 0.001
Complete median sternotomy	1095 (79.9%)	551 (96.3%)	
Right thoracotomy	275 (20.1%)	21 (3.7%)	
Preoperative oxygen saturation (%)	98.0 [97.0, 99.0]	96.0 [90.0, 98.0]	< 0.001
Oxygen saturation (%)	98.0 [97.0, 99.0]	98.0 [96.0, 99.0]	< 0.001
**Trajectory of SBP change**			
Cluster 1	931 (67.4%)	340 (58.4%)	< 0.001
Cluster 2	349 (25.3%)	40 (6.9%)	< 0.001
Cluster 3	102 (7.4%)	202 (34.7%)	< 0.001
**Trajectory of DBP change**			
Cluster 1	83 (6.0%)	31 (5.3%)	1.890
Cluster 2	1032 (74.7%)	310 (53.3%)	< 0.001
Cluster 3	267 (19.3%)	241 (41.4%)	< 0.001
**Trajectory of MAP change**			
Cluster 1	229 (16.6%)	234 (40.2%)	< 0.001
Cluster 2	826 (59.8%)	265 (45.5%)	< 0.001
Cluster 3	18 (1.3%)	44 (7.6%)	< 0.001
Cluster 4	309 (22.4)	39 (6.7%)	< 0.001
**SBP variability**			
Overall slope	1.9 [1.6, 2.3]	1.6 [1.4, 1.9]	< 0.001
Overall coefficient of variation	0.3 [0.2, 0.3]	0.3 [0.2, 0.3]	0.437
Pre-slope	1.7 [1.3, 2.3]	1.5 [1.1, 2.1]	< 0.001
Intra-slope	1.6 [1.1, 2.1]	1.3 [1.0, 1.8]	< 0.001
Post-slope	1.9 [1.5, 2.4]	1.7 [1.3, 2.1]	< 0.001
Pre-coefficient of variation	0.1 [0.1, 0.2]	0.1 [0.1, 0.2]	0.326
Intra-coefficient of variation	0.2 [0.2, 0.3]	0.2 [0.2, 0.3]	0.013
Post-coefficient of variation	0.1 [0.1, 0.2]	0.1 [0.1, 0.2]	0.006
**DBP variability**			
Overall slope	1.2 [1.0, 1.4]	1.1 [0.9, 1.3]	< 0.001
Overall coefficient of variation	0.2 [0.2, 0.2]	0.2 [0.2, 0.3]	< 0.001
Pre-slope	1.2 [0.9, 1.6]	1.0 [0.7, 1.3]	< 0.001
Intra-slope	1.2 [0.8, 1.5]	1.1 [0.8, 1.4]	0.131
Post-slope	1.0 [0.7, 1.3]	1.0 [0.7, 1.3]	0.539
Pre-coefficient of variation	0.1 [0.1, 0.2]	0.1 [0.1, 0.2]	0.167
Intra-coefficient of variation	0.2 [0.1, 0.2]	0.2 [0.2, 0.3]	< 0.001
Post-coefficient of variation	0.1 [0.1, 0.1]	0.1 [0.1, 0.2]	< 0.001
**MAP variability**			
Overall slope	1.5 [1.3, 1.7]	1.3 [1.1, 1.5]	< 0.001
Overall coefficient of variation	0.2 [0.2, 0.3]	0.2 [0.2, 0.3]	0.033
Pre-slope	1.5 [1.1, 1.9]	1.2 [0.9, 1.6]	< 0.001
Intra-slope	1.3 [1.0, 1.7]	1.2 [0.9, 1.6]	0.004
Post-slope	1.3 [1.0, 1.7]	1.2 [0.9, 1.6]	< 0.001
Pre-coefficient of variation	0.2 [0.1, 0.2]	0.2 [0.1, 0.2]	0.051
Intra-coefficient of variation	0.2 [0.1, 0.2]	0.2 [0.2, 0.3]	< 0.001
Post-coefficient of variation	0.1 [0.1, 0.1]	0.1 [0.1, 0.2]	< 0.001

*ASD* atrial septal defect, *VSD* ventricular septal defect, *PDA* patent ductus arteriosus, *PFO* patent foramen ovale, *TOF* tetralogy of fallot, *TAPVC* total anomalous pulmonary venous connection, *LOS* length of stay, *SBP* systolic blood pressure, *DBP* diastolic blood pressure, *MAP* mean arterial pressure.

### Data-driven clusters group blood pressure time-series data

**Figure 2:**
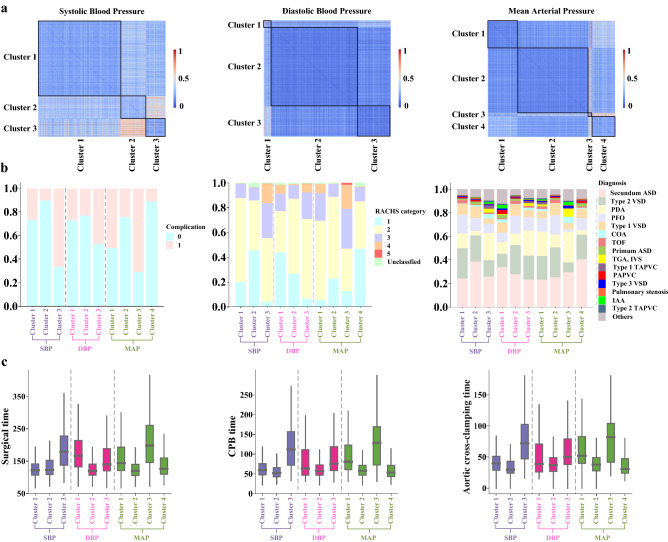
Clustering of intraoperative blood pressure and comparison of other characteristics between clusters. **(a)** Similarity matrix of blood pressure where rows and columns were ordered by a partition detected through k-means clustering. **(b)** Composition of each cluster in terms of whether the patients experienced postoperative complications, risk categories of operation, or primary diagnoses. **(c)** Distribution of surgical time, CPB time, or aortic cross-clamping time between each cluster. In the box plots, box edges represent the 25th and 75th percentiles, the centerline shows the median and whiskers extend from the box edges to the 1.5× interquartile range and *means P < 0.001 (Kruskal–Wallis H-test).

When patients were ordered by their blood pressure cluster, the block-like structure of the similarity matrix becomes evident (Fig. 2a). Black lines along the diagonal marked blocks of patients grouped into the same cluster and similarities between patients in the marked blocks have some differences from those outside the mark blocks. We also compared the composition of each cluster in terms of whether the patients experienced postoperative complications, risk categories of operation, or primary diagnoses (Fig. 2b). Notably, there were significant differences in these components of clusters, and the subjects belonged to clusters with higher rates of complications harbored more complex surgery. Clusters with higher rates of complications were also composed primarily of patients with high levels of patent ductus arteriosus, coarctation of aorta, and type 1 total anomalous pulmonary venous connection, which have a higher risk than other diagnoses. In addition, we compared surgical time, CPB time, and aortic cross-clamping time between clusters and found statistically significant differences between clusters for these times (Fig. 2c). The mean and 95% confidence interval of the blood pressure readings during the surgery between different clusters were shown in Supplementary Fig. S3.

### Performance of the complication prediction model

**Figure 3:**
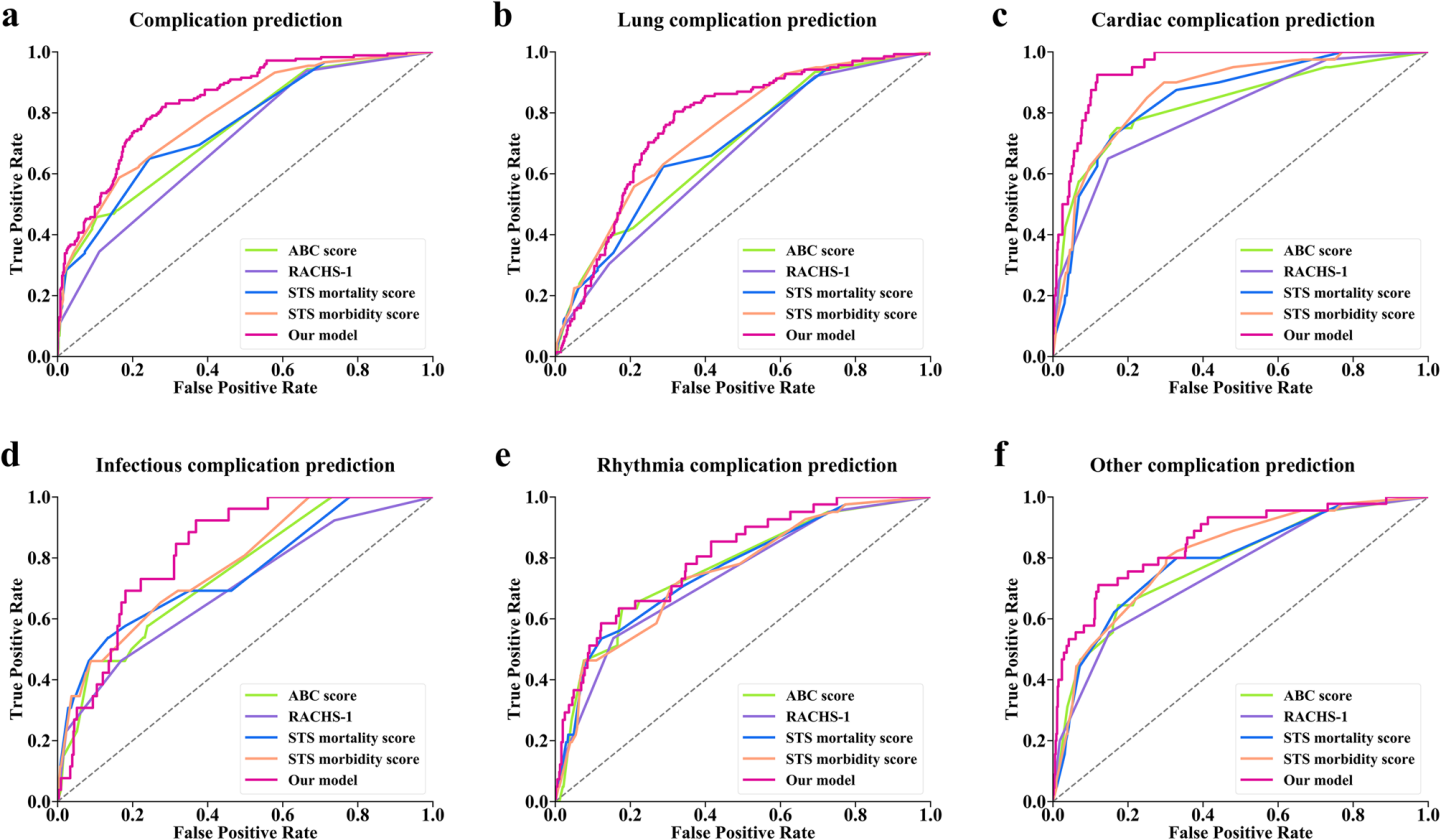
Receiver operating characteristic curve of the predictive models in the test data set. **(a)** Binary classification; **(b–f)** Multi-label classification. *ABC* Aristotle basic complexity, *RACHS-1* risk adjustment for congenital heart surgery, *STS* Society of Thoracic Surgeons.

To test the potential of our model to aid postoperative complication prediction we evaluated the performance of our proposed model and four consensus-based risk models using receiver operating characteristic curves and other evaluation metrics (Fig. 3, Table 2, Supplementary Table S2). We found that for both the binary and multi-label classification tasks, the predictions made by our model are considerably more accurate than the predictions made by consensus-based risk models. The STS-EACTS morbidity score performed relatively well in both types of classification tasks compared with other risk models. Cardiac complication prediction has a higher AUC of 0.946, whereas lung complication prediction has a lower AUC of 0.785 (Fig. 3b–f, Supplementary Table S2).

**Table 2:** Experimental results of binary classification and multi-label classification on the test set.

	Binary classification				Multi-label classification			
	ACC	Recall	F1	AUC	ACC	Micro-Recall	Micro-F1	Macro-AUC
Our method	0.756	0.791	0.661	0.839	0.831	0.703	0.451	0.850
ABC score	0.732	0.480	0.518	0.745	0.761	0.541	0.308	0.763
RACHS-1	0.725	0.345	0.430	0.705	0.805	0.438	0.308	0.730
STS mortality score	0.746	0.407	0.490	0.755	0.829	0.459	0.345	0.770
STS morbidity score	0.736	0.627	0.587	0.792	0.729	0.652	0.322	0.789

### Inspection of model features

**Figure 4:**
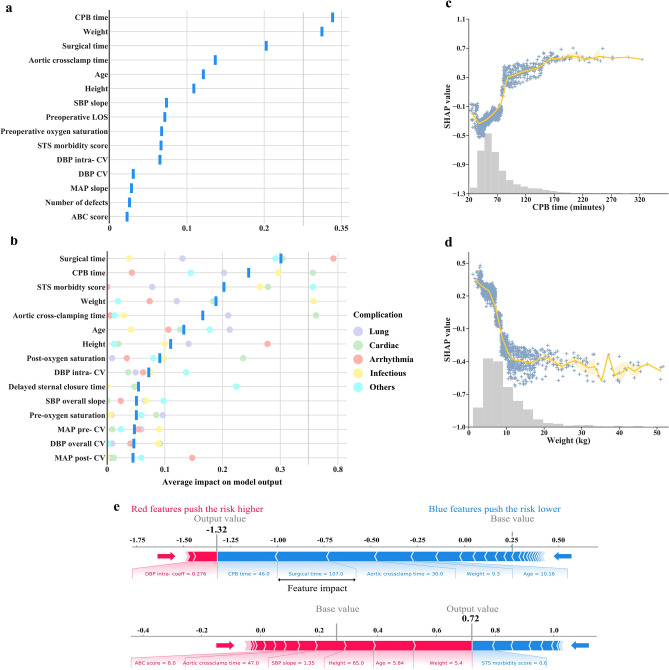
Feature inspection. **(a,b)** Importance estimates assigned by our proposed method to the top 15 features in both the binary **(a)** and multi-label **(b)** classification. The importance of features in multi-label classification was measured as the averaged feature importance estimated by five categories of complication prediction, and the feature importance of each category prediction was represented by different colored circles. **(c,d)** Scatter plots showing the relationship between the varying feature and SHAP value for CPB time **(c)** and patient weight **(d)**. The gray histogram shows the distribution of values for that feature in the training set. The orange line and shade represent the mean and the 95% confidence interval of the regression line. **(e)** Visualization of two individual examples for the explanation risk of postoperative complications. Each feature contributes to pushing the model output from the base value (the average model output over the training dataset we passed) to the model output. Red features mean pushing the prediction higher risk and blue features pushing the prediction lower risk.

In Fig. 4a,b, we list the top 15 features by mean absolute SHAP value for both the binary and multi-label classification, and different colored circles in Fig. 4b represent the feature importance of each category in multi-label classification. The top 15 features importance of each complication category are respectively shown in Supplementary Fig. S4. The relationship between feature value and SHAP value in binary classification is illustrated in more detail for the features in Fig. 4c,d, with further examples in Supplementary Fig. S5. When removing the top 15 features in turn, the removal of CPB time noticeably decreased model performance in the binary classification, as is also observed in the analysis based on SHAP values. While these analyses show the overall effect of the features, SHAP values can also be inspected for individual predictions to identify the influential features (see Fig. 4e). All these effects explain why the model predicted a specific risk and thus allow appropriate interventions before deleterious outcomes ensue.

## Discussion

We developed an efficient machine-learning-based model that comprehensively integrated patient- and surgery-specific static features and intraoperative time-series features to predict postoperative complications before they occur. Based on a comparison with existing consensus-based risk models, our model achieves superior performance both in binary and multi-label classification. Different from the traditional black-box model, we used the XGBoost model and SHAP framework which take advantage of artificial intelligence to process complex and high-dimensional features and identify the quantitative association between factors and prediction result to explain the prediction at different levels. In addition, we introduced blood pressure variability and k-means algorithm combined with soft-DTW to preprocessing intraoperative blood pressure. Such an approach can improve the interpretability of intraoperative time-series features, and we know which phase of fluctuation in surgery is more likely to lead to complications. This combination of model performance and interpretability allows physicians to receive the best predictions while also gaining insight into why those predictions were made.

The risk profiles learned by our model are clinically relevant. First, accumulating evidence has demonstrated the prolonged duration of CPB as a risk factor for neurologic, respiratory, infective, and renal complications. However, CPB time is frequently dichotomized at heterogeneous time points or the association between duration and risk of complication was not well characterized. Yamauchi and colleagues identified CPB times > 5 h as a risk factor of postoperative acute kidney injury^19^. Agarwal and colleagues reported that a longer CPB time was significantly associated with a great number of cardiac and extracardiac complications^13^. In our study, CPB time is also the most important variable as is observed in the analysis based on SHAP values and selection of variables guided by model performance. We provide a more useful perspective by considering the quantitative effect of continuous CPB time and its relationship with complications (Fig. 4c). The actionable knowledge such as control the CPB time under 80 min or 160 min will relatively control the risk of postoperative complications at different levels can be generated from these explainable plots. Second, clinical surgeons can now quantify risks of postoperative complications adjusted for other factors to the younger, those who are low weight and more susceptible to the environment. The exact relationships described in Fig. 4 and Supplementary Fig. S5 clearly show the patterns and threshold points for the risk. Third, different diagnoses are associated with different risk levels of postoperative complications. For example, patent ductus arteriosus or tetralogy of fallot patients may be more critically ill as they have more postoperative complications when compared with patients with other defects. When considering the standardization of care to reduce unwanted clinical deterioration, these data suggest that resources need to be differentially deployed to address differential rates of complications.

To the best of our knowledge, before, during, and after CPB are 3 distinctly different phases in cardiac surgery, and changes in blood pressure at these 3 phases may also have different effects on complications^20^. Due to the lack of our data on patients’ start and end times of CPB, we attempted to explore the impact of changes in blood pressure at different phases of surgery distinguished by changes in temperature^21^. Naturally, changes in blood pressure before hypothermia had a minimal effect on the risk of complications when compared with intra- and post- hypothermia (Fig. 4, Supplementary Fig. S4). Interestingly, we found that the smaller average slope of systolic blood pressure was associated with an increased risk of postoperative complications in both the univariable analysis and prediction model (Table 1, Supplementary Fig. S5). This finding stands in contrast to the common belief that rapid fluctuations in intraoperative arterial blood pressure are deleterious and that clinicians should strive to maintain ‘railroad track’ hemodynamics^22^. One possible interpretation may be that patients with shorter surgical times quickly have steep changes in blood pressure readings because the trends in blood pressure readings are all from normal to lower and finally back to normal. For the analysis of postoperative outcomes, the complexity of cardiac surgery was considered an important risk factor in other studies^13^. The length of surgical time indirectly reflects the complexity of the surgery.

When using this model to generate early warnings before complications occur, it is important to understand the balance between recall (the sensitivity) and precision. Given that the ratio of positive to negative in our data is unbalanced, especially for specific complication types, we adjust one weight parameter in the model which can control the balance of positive and negative weights. However, in the multi-label classification, infectious and rhythm complications achieved the worst F1 score (Supplementary Table S2) when compared with other types of complications. One possible interpretation may be that the ratio of positive and negative for this type of complication is too unbalanced and adjusting the weight parameter to improve recall will result in a significant reduction in precision. In addition, the correlation between different types of complications and features is not the same, the current features maybe not the strongest predictor of infectious and rhythm complications (Supplementary Fig. S4).

The field of medicine is full of data science challenges that have the potential to fundamentally affect the way medicine is practiced. More and more data-driven predictions of patient prognosis are being proposed. However, black-box models which did not provide any explanations about why make this prediction, are difficult for physicians to trust. The ability to establish which features contributed to a prediction ensures that this technology remains interpretable to its clinical users. Using SHAP values, we see that the model provided quantitative insight into the exact changes in risk caused by changes in the features of certain patients. In addition, the interpretable prediction made by our model is easy for physicians to trust and provide insight into how they should respond before the condition worsens.

Even though our model gets a better performance when compared with other consensus-based risk models, it should still be considered as an initial attempt. In the multi-label predictions, considering the low number of some complication cases, we classified complications into five complication classes rather than predicting specific types of complications. To enhance the clinical availability, future attempts can focus on predicting specific types of complications and identifying features that led to this risk. Another future enhancement would be the integration of abundant preoperative data, such as detailed laboratory results of patients into the prediction model. More high-fidelity intraoperative data such as heart rate, End-tidal CO_2_, and respiratory rate could include in the prediction model, thus potentially leading to more accurate predictions.

There are some possible limitations in this study. We only used relatively few data (n = 1964) from a single center to train and validate the model; thus, multicenter data will be used to train and validate the model. While we believe that a specific model trained on data from a single center will give a more specific prediction for a single center. This approach can be used to train specific models for data from multi-centers when considering hospitals and surgeons as features. In addition, we also performed a time-based split to divide the dataset into separate training and test sets (the experimental results of binary classification and multi-label classification on the time-based test set is listed in Supplementary Table S3). In shortly, the performance of proposed model is better than the risk adjustment models especially gave a much higher recall. Compared with randomly dividing the training sets and test sets, the time-based split prediction results have decreased slightly. One possible interpretation may be that the treatment of complex defects has greatly improved with the rapid development of surgical and interventional treatment, so there may be some differences in the characteristics of the dataset from year to year. Another limitation is the low frequency at which blood pressure was obtained, specifically one measurement every 5 min to 10 min during the surgery. A narrower time interval would have been desirable and possibly more illuminating.

## Conclusions

In summary, with a novel interpretable machine learning algorithm, we can predict whether a patient has the complication after congenital heart surgery and what kind of complications will occur and explain the specific patient characteristics that led to this prediction. This prediction model achieved higher accuracy and sensitivity compared to risk adjustment models. We believe the combination of model performance and interpretability could provide useful information for physicians and can be used as part of clinical decision making.

## Methods

### Study design and population

A total of 2858 pediatric patients who underwent congenital heart surgery between December 2015 and December 2018 at the Children’s Hospital of Zhejiang University School of Medicine were enrolled in the present analysis. Exclusion criteria included patients who died during the surgery, patients who lacked intraoperative anesthesia records, or patients who underwent surgery without CPB, for which the selection process of eligible participants is shown in Supplementary Fig. S1. Thus, the dataset from the remaining 1964 patients were included in the present analyses. This retrospective study was performed according to relevant guidelines and approved by the institutional review board of the Children’s Hospital, Zhejiang University School of Medicine with a waiver of informed consent (2018_IRB_078).

### Data collection and pre-processing

The following data elements were requested: gender, age, height, and weight of patients; diagnoses and types of procedures; surgical time, CPB time and aortic cross-clamping time; surgical access route; preoperative and postoperative oxygen saturation; intraoperative anesthetic record data; and postoperative complications.

The most challenging part of the data preprocessing is the time-series vital signs data during surgery with different lengths which cannot be directly used to construct the prediction model. The evidence-based literature supporting temperature management in cardiac surgery suggests that mild (32–35 °C), moderate (28–32 °C), or deep hypothermic (< 28 °C) is used to protect the brain and other vital organs during cardiopulmonary bypass^21^. Firstly, we divided surgery into three phases according to the changes in temperature, namely, the pre- (normal temperature—35 °C), intra- (< 35 °C), and post- (35 °C—normal temperature) hypothermic periods. Blood pressure variability including the coefficient of variation and slope was used to measure blood pressure fluctuations of different phases of surgery (Fig. 1). The coefficient of variation was defined as the standard deviation divided by the mean of each blood pressure sequence. In addition, the average changes (the slope) were also calculated as follows:

$${\text{Slope}} = \frac{1}{{N - 1}}\sum\limits_{{k = 1}}^{{N - 1}} {\frac{{|BP_{{k + 1}} - BP_{k} |}}{{t_{{k + 1}} - t_{k} }}}.$$

To further capture the dynamic temporal pattern of blood pressure during surgery, we used a k-means algorithm to cluster the pattern of blood pressure changes in distinct trajectories (Fig. 1). In time-series analyses, the smoothed formulation of dynamic time warping (soft-DTW) was used to measure the similarity between two temporal sequences, which may vary in length and speed^23^. To perform clustering of the blood pressure, we constructed a matrix R whose elements R_i,j_ equal the blood pressure trajectory similarity calculated by soft-DTW between patient i and patient j. Next, we performed k-means clustering on the similarity matrix R and the number of clusters was determined by maximizing the average silhouette coefficient and minimizing the within clusters sum of squares (a more detailed description of determining the optimal number of clusters is illustrated in Supplementary Fig. S2). The application of k-means clustering was applied after data splitting when training prediction model. For each patient in the test set, we computed the similarities between data points and all centroids and assigned each data point to the closest cluster. Collectively, the extracted items including blood pressure variability and clustered trajectories combined with patient characteristics were summarized into 45 features (detail shown in Table 1), which were subsequently used to construct the machine learning prediction model.

The missing values were imputed using multivariate imputation via chained equations package in R^24^. It is a practical approach to generating imputations based on a set of imputation models, one for each variable with missing values. We used the random forest to fit regression trees of the data and imputed each missing value as the prediction based on trees. Class imbalance is also a problem in this study since the number of patients with postoperative complications is relatively small in compassion with the number without complications in some scenarios. It is important to properly adjust your metrics and methods to adjust for your goals^25^. In this study, we used the scale_pos_weight hyperparameter in XGBoost which is designed to tune the behavior of the algorithm for imbalanced classification problems. It has the effect of weighing the balance of positive examples, relative to negative examples when boosting decision trees.

### Postoperative complication labels

The label of whether the patient had any complications after surgery and what kind of complications occurred was collected by clinicians based on the review of medical records. Based on more than 30 defined complications (detailed definition of the types of complications is listed in Supplementary Table S1), we classified complications into five complication classes: lung complication, cardiac complication, rhythm complication, infectious complication, and other complications^26^. Cardiac complication indicates that a complication symptom appeared in the heart except for arrhythmia, such as cardiac dysfunction resulting in low cardiac output, pulmonary hypertension, and so on. Rhythm complication indicates that any cardiac rhythm other than normal sinus rhythm. Infectious complication is defined as the successful invasion and growth of organisms in the tissues of the host such as sepsis, urinary tract infection, and wound infection. Other complications indicate that the symptoms of complications in other organs apart from the lung and heart such as thrombosis, liver dysfunction, ascites, and so on. It is worth mentioning that a patient can experience multiple postoperative complications. In this study, we defined two tasks, binary classification and multi-label classification, to predict whether the corresponding patient has complications and what kind of complications.

### Statistical analysis

The patients were categorized according to whether they had experienced postoperative complications. Categorical variables were presented as counts and percentages, and continuous variables as median with interquartile range (IQR) as 25th and 75th percentiles. The Chi-square test was used to compare categorical variables of patients with and without this outcome, and the continuous variables were compared using the Mann–Whitney U test. Bonferroni’s correction was used to control the family-wise error rate when multiple comparisons were performed. All tests were two-sided, and statistical significance was set at P-value < 0.05 for all analyses. Data analyses were performed using the published package in the Python (version 3.7) programming environments.

### Model development and evaluation

As the collected features may have a variety of nonlinear interactions, we used XGBoost, a scalable tree boosting system, to link input features with postoperative complications. It implements machine learning algorithms under the gradient boosting framework and provides a parallel tree boosting that solves many data science problems in a fast and accurate way^27^. To understand how single features relate to the model output we used SHAP (Shapley Additive exPlanations) values, which are suited for complex models such as neural networks and gradient-boosting machines^28^. The impact of each feature on the model is represented using Shapley values, which are from the game theory and provide a theoretically justified method for allocation of a coalition’s output among the members of the coalition^28^.

To ensure stability and extrapolation of machine learning model, we randomly divided the dataset into separate training (n = 1375) and test sets (n = 589) at a ratio of 7:3. We used fivefold cross-validation on the training set to tune hyperparameters for each classification and evaluated the final performance using the independent test set. The optimal model parameters were determined in a random search of 500 different combinations of hyperparameters of XGBoost. For the final binary classification model, we used learning rate as 0.01, gradient boosted trees as 292, maximum tree depth as 3, and minimum child weight of any branch in the trees as 5. For the final multi-label classification model, these parameters respectively were 0.02, 140, 5, and 4.

The accuracy, area under the receiver operating characteristic curve (AUC), recall, and F1 score were the metrics used to evaluate binary classification performance. The accuracy, micro-recall, micro-F1 score, and macro-AUC were the metrics used to evaluate multi-label classification performance. The F1 score is a measure of test data accuracy, which is a weighted average between precision and recall. The micro average calculates metrics globally by counting the total true positives, false negatives, and false positives; while the macro average calculates metrics for each label and finds their unweighted mean. We compared the performance of our prediction model with four risk adjustment models mentioned above in the binary and multi-label classification. For patients undergoing multiple procedures, the procedure with the highest level was scored. We assessed the RACHS-1 category, the ABC score, the STS-EACTS mortality and morbidity score as a predictor of postoperative complications by using the univariable logistic regression respectively.

### Ethics declarations

This study was approved at 2018-09-19 by the Institutional Review Board/Ethics Committee of the Children’s Hospital, Zhejiang University School of Medicine (2018_IRB_078). Written informed consent was waived by the Institutional Review Board/Ethics Committee, as the utilization of anonymized retrospective data does not require patient consent under the local legislation.

## Supplementary Information

Supplementary Information.

## Data Availability

Data collected for this study are highly sensitive, and if reasonably requested, data supporting the findings of this study can be obtained from the corresponding author on reasonable request.
