# Supplementary material for: Explainable machine-learning predictions for complications after pediatric congenital heart surgery
Source: Sci Rep. 2021 Aug 26;11:17244. doi: 10.1038/s41598-021-96721-w (PMC8390484; doi:10.1038/s41598-021-96721-w)
Supplement: Supplementary file 1 — Supplementary Information. [file 41598_2021_96721_MOESM1_ESM.pdf]

Explainable machine-learning predictions for complications after pediatric congenital heart surgery

Xian Zeng[1,2], Yaoqin Hu[1], Liqi Shu[3], Jianhua Li[1], Huilong Duan[2], Qiang Shu[1*]; Haomin Li[1*]

1. The Children's Hospital, Zhejiang University School of Medicine, National Clinical Research Center for Child Health, Hangzhou, China
2. The College of Biomedical Engineering and Instrument Science, Zhejiang University, Hangzhou, China
3. Department of Neurology, Rhode Island Hospital, Brown University, Providence, United States

Legends:

Table S1. Definition of postoperative complications**.**

Table S2. Experimental results of different categories in multi-label classification on test set.

Table S3. Experimental results of binary classification and multi-label classification on the time-based split test set.

Table S4. Evaluation of interpretability frameworks on our dataset.

Fig. S1. Flowchart of selection process of eligible participants included in this analysis.

Fig. S2. Determining the optimal number of clusters in k-means clustering.

Fig. S3. Mean and 95% confidence interval of blood pressure readings of different clusters.

Fig. S4. Averaged feature importance estimates by our proposed method for each category of complication.

Fig. S5. The influence of varying top 15 features values at different phases on complication prediction.

Supplementary tables

Supplementary Table S1. Definition of postoperative complications

Complication	Definition	Count (%)
**Cardiac**	A symptom appeared in the heart except for arrhythmias, such as cardiac dysfunction resulting in low cardiac output, and pulmonary hypertension.	134 (23.0%)
Cardiac dysfunction resulting in low cardiac output	Low cardiac output state, characterized by use of 3 inotropes and associated with the following: tachycardia, oliguria, decreased skin perfusion, need for increased inotropic support (10% above baseline at admission), metabolic acidosis, widened Arterial – Venous oxygen saturation, need to open the chest, or need for mechanical support.	117 (20.1%)
Pulmonary hypertension	Clinically significant elevation of pulmonary arterial pressure, requiring intervention. Typically, the mean pulmonary arterial pressure is greater than 25 mmHg in the presence of a normal pulmonary arterial occlusion pressure	12 (2.1%)
Pulmonary hypertension (PA pressure > systemic pressure)	Clinically significant elevation of pulmonary arterial pressure, requiring intervention, with the pulmonary arterial pressure being greater than the systemic arterial pressure (supra-systemic pulmonary arterial pressure)	7 (1.2%)
Postoperative/Postprocedural mechanical circulatory support	Utilization of postoperative/postprocedural mechanical support, of any type (IABP, VAD, ECMO, or CPS), for resuscitation/CPR or support, during the postoperative/postprocedural time period.	7 (1.2%)

(IABP, VAD, ECMO, or CPS)		
Pericardial effusion, Requiring drainage	Abnormal accumulation of fluid in the pericardial space, Requiring drainage, By any technique	5 (0.9%)
Pulmonary vein obstruction	Clinically significant stenosis or obstruction of pulmonary veins. Typically diagnosed by echocardiography or cardiac catheterization, this may present with or without symptoms	3 (0.5%)
Endocarditis-postprocedural infective endocarditis	An infection of the endocardial surface of the heart, which may include one or more heart valves, the mural endocardium, or a septal defect	2 (0.3%)
**Rhythm**	Any cardiac rhythm other than normal sinus rhythm	131 (22.5%)
Arrhythmia	Any cardiac rhythm other than Normal Sinus Rhythm	84 (14.4%)
Arrhythmia necessitating pacemaker, Temporary pacemaker	Implantation and utilization of a temporary pacemaker for treatment of any arrhythmia including heart block (atrioventricular [AV] heart block)	56 (9.6%)
**Lung**	A symptom appeared in the lung such as pneumonia, pleural effusion, and pneumothorax.	432 (74.2%)
Pneumonia	A respiratory disease characterized by inflammation of the lung parenchyma (including alveolar spaces and interstitial tissue), most commonly caused by infection	241 (41.4%)
Pleural effusion, Requiring drainage	Abnormal accumulation of fluid in the pleural space, Requiring drainage, By any technique.	107 (18.4%)
Pneumothorax, Requiring	A collection of gas in the pleural space resulting in collapse of the lung on the affected side. Requiring drainage	97 (16.7%)

drainage	by chest tube or thoracocentesis	
Atelectasis	Atelectasis is the collapse of part or all of a lung by blockage of the bronchus or bronchioles leading to retraction of the lung and an airless state Chest radiographs and/or CT scans show displacement of fissures, opacification of the collapsed lobe, displacement of the hilum, mediastinal shift toward the side of collapse, loss of volume on ipsilateral hemithorax, elevation of ipsilateral diaphragm, crowding of the ribs, compensatory hyperlucency of the remaining lobes, and silhouetting of the diaphragm or the heart border.	78 (13.4%)
Postoperative/Postprocedural respiratory insufficiency requiring reintubation	Reintubation required after initial extubation. In other words, the need to reinstitute postoperative or postprocedural mechanical ventilation after a planned extubation and prior to discharge, or after a planned extubation and after discharge but within 30 days of surgery	43 (7.4%)
Postoperative respiratory insufficiency requiring mechanical ventilatory support >7 days	Respiratory Insufficiency requiring mechanical ventilatory support from surgery or procedure to greater than 7 days postoperatively	12 (2.1%)
Chylothorax	Presence of lymphatic fluid in the pleural space, commonly secondary to leakage from the thoracic duct or one of its main tributaries. Thoracocentesis is the gold standard for diagnosis and generally reveals a predominance of lymphocytes and/or a triglyceride level greater than 110 mg/dL	10 (1.7%)
Respiratory failure, Requiring tracheostomy	Failure to wean from mechanical ventilation necessitating the creation of a surgical airway	1 (0.2%)

**Infectious**	The successful invasion and growth of organisms in the tissues of the host, such as sepsis, urinary tract infection and wound infection	90 (15.5%)
Sepsis	Sepsis is defined as "evidence of serious infection accompanied by a deleterious systemic response". In the time period of the first 48 postoperative or postprocedural hours, the diagnosis of sepsis requires the presence of a Systemic Inflammatory Response Syndrome (SIRS) resulting from a proven infection (such as bacteremia, fungemia or urinary tract infection)	82 (14.1%)
Urinary tract infection	A urinary tract infection is an infection of the urinary tract, as defined by positive urine culture or white blood cells (WBCs) present on urinalysis. A urinary tract infection that will be counted as an operative or procedural complication must occur prior to hospital discharge or after hospital discharge but within 30 days of the procedure.	6 (1.0%)
Wound infection	Erythema, possible induration and possible fluctuance of a surgical wound with possible drainage and possible tissue separation. Though wound cultures may be positive, this is not an absolute requirement for establishing this clinical diagnosis.	3 (0.5%)
**Other**	The symptoms of complications in other organs apart from the lung and heart, such as thrombosis, liver dysfunction, and ascites	155 (26.6%)
Thrombosis	Acute or chronic occlusion of a blood vessel by thrombus, embolus, tumors, external compression, trauma, hypercoagulable states, or other cause	59 (10.1%)

Sternum left open	Sternum was left open postoperatively (i.e. planned or unplanned). The goal is for delayed sternotomy closure	41 (7.0%)
Liver dysfunction	Dysfunction of the liver that results in hypoalbuminemia (<2 grams/dL), coagulopathy (PT>1.5×upper limits of normal), and hyperbilirubinemia (>3.0×upper limits of normal). Select this complication if the patient develops 2 out of these 3 laboratory abnormalities	25 (4.3%)
Ascites	Accumulation of fluid in the peritoneal cavity	14 (2.4%)
Acute renal failure requiring temporary dialysis with the need for dialysis not present at hospital discharge	New onset oliguria with sustained urine output<0.5 cc/kg/hr for 24 hours and creatinine>1.5 times upper limits of normal for age (or twice the most recent values), with eventual need for dialysis or hemofiltration. The patient does not require dialysis at the time of hospital discharge or death in the hospital	11 (1.9%)
Reoperation during this admission (unplanned reoperation)	Any additional unplanned operation prior to discharge	9 (1.5%)
Neurological deficit persisting at discharge	Newly recognized and/or newly acquired deficit of neurologic function leading to inpatient referral, therapy, or intervention not otherwise practiced for a similarly unaffected inpatient, With a persisting neurologic deficit present at hospital discharge	5 (0.9%)
Multi-System Organ Failure	Multi-System Organ Failure (MSOF) is a condition where more than one organ system has failed (for example, respiratory failure requiring mechanical ventilation combined with renal failure requiring dialysis)	4 (0.7%)
Paralyzed diaphragm (possible	Presence of elevated hemi-diaphragm(s) on chest radiograph in conjunction with evidence of weak, immobile,	2 (0.3%)

phrenic nerve injury)	or paradoxical movement assessed by ultrasound or fluoroscopy	
Seizure	The clinical and/ or electroencephalographic recognition of epileptiform activity.	1 (0.2%)
Other complication	Any complication not otherwise specified in this list	20 (3.4%)
**Operative mortality**		30 (1.5%)

Supplementary Table S2. Experimental results of different categories in multi-label classification on test set

Category	ACC	Recall	F1	AUC
Lung	0.710	0.797	0.563	0.764
Cardiac	0.908	0.775	0.534	0.946
Rhythm	0.868	0.689	0.443	0.859
Infectious	0.814	0.692	0.247	0.824
Other	0.934	0.489	0.530	0.856

Supplementary Table S3. Experimental results of binary classification and multi-label classification on the time-based split test set

	Binary Classification				Multi-label Classification			
	ACC	Recall	F1	AUC	ACC	Micro-Recall	Micro-F1	Macro-AUC
**Our method**	**0.740**	0.705	**0.628**	**0.780**	**0.845**	**0.621**	**0.395**	**0.810**
**ABC score**	0.723	0.410	0.479	0.716	0.771	0.513	0.268	0.704
**RACHS-1**	0.503	**0.929**	0.537	0.682	0.801	0.300	0.197	0.638
**STS mortality score**	0.671	0.607	0.534	0.723	0.836	0.358	0.247	0.732
**STS morbidity score**	0.689	0.568	0.532	0.730	0.784	0.533	0.287	0.749

Supplementary Table S4. Evaluation of interpretability frameworks on our dataset

	SHAP	LIME
**Identity**	100%	100%
**Separability**	100%	100%
**Difference**	3.11	3.89

For each framework, we normalize the explanations and original dataset, then calculate the mean pairwise Euclidean distances separately. To measure the identity metric, if the distance between the two instances is equal to zero, then the distance between their explanations should be equal to zero. To measure the separability metric, we choose a subset S of the testing data set that has no duplicates and get their explanations. Then for every instance s in S, we compare its explanation with all other explanations of instances in S and if such explanation has no duplicate then it

satisfies the separability. To measure difference metric, we calculate the difference value between two distance matrices, then calculate the mean of the absolute value of the difference. The smaller the difference, the more similar the two matrices are.

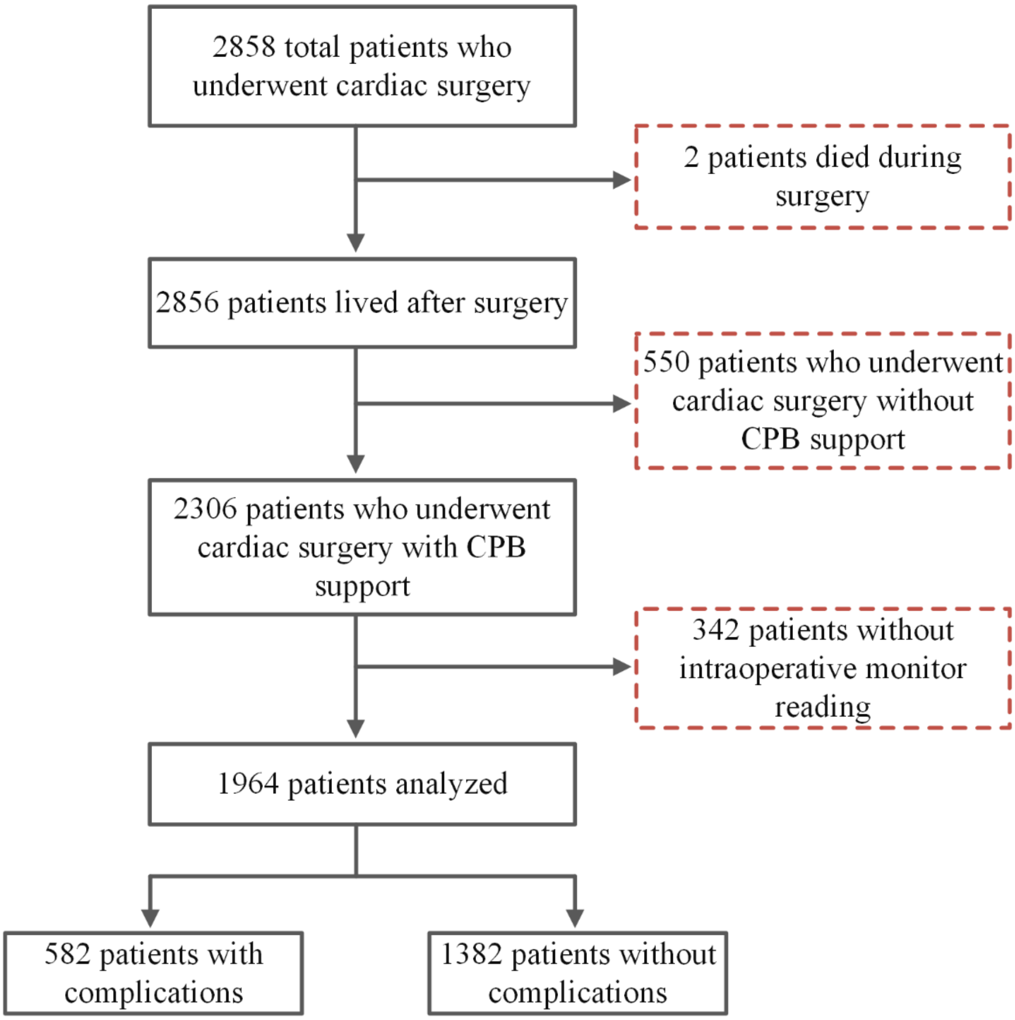

Supplementary Fig. S1. Flowchart of selection process of eligible participants included in this analysis.

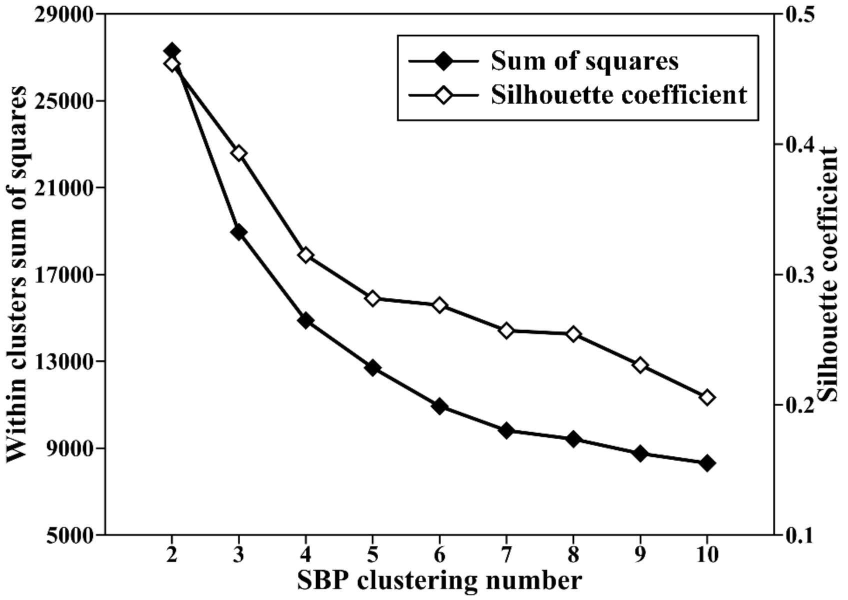
Sum of squares
Silhouette coefficient
Within clusters sum of squares
Silhouette coefficient
29000
25000
21000
17000
13000
9000
5000
0.5
0.4
0.3
0.2
0.1
2 3 4 5 6 7 8 9 10
SBP clustering number

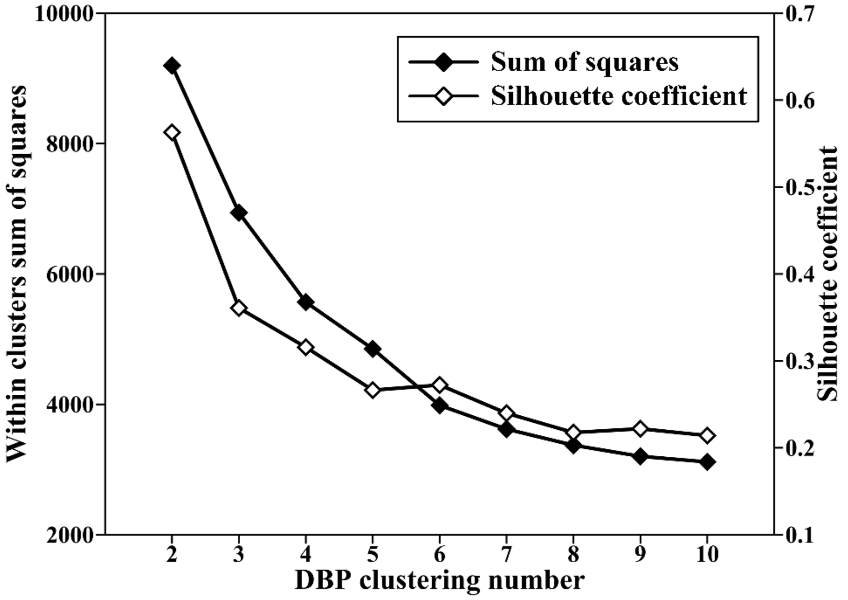
Sum of squares
Silhouette coefficient
Within clusters sum of squares
Silhouette coefficient
10000
8000
6000
4000
2000
0.7
0.6
0.5
0.4
0.3
0.2
0.1
2 3 4 5 6 7 8 9 10
DBP clustering number

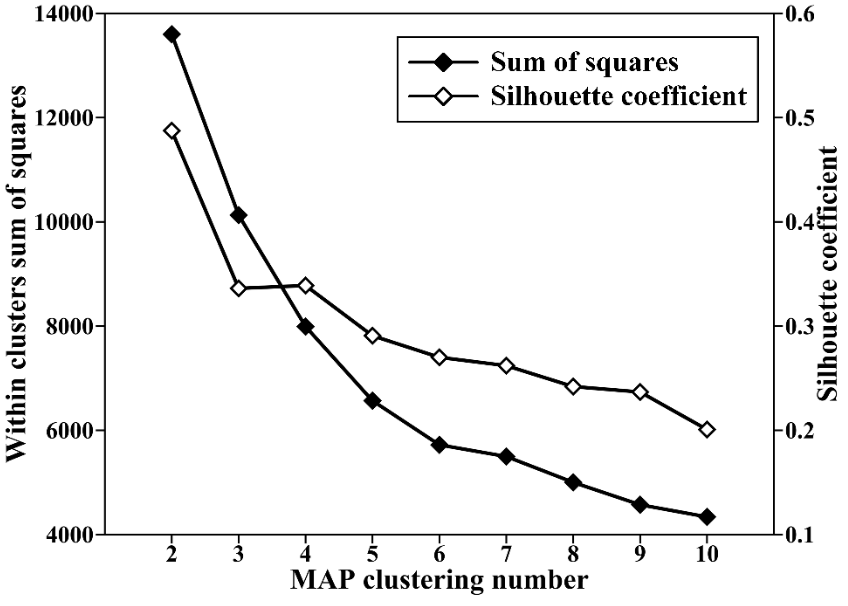
Sum of squares
Silhouette coefficient
Within clusters sum of squares
Silhouette coefficient
14000
12000
10000
8000
6000
4000
0.6
0.5
0.4
0.3
0.2
0.1
2 3 4 5 6 7 8 9 10
MAP clustering number

Supplementary Fig. S2. Determining the optimal number of clusters in k-means clustering. The silhouette coefficient is a measure of how similar an object is to its own cluster compared to other clusters, a high value indicates that the object is well matched to its own cluster and poorly matched to neighboring clusters. In the clustering of systolic blood pressure, diastolic blood pressure and mean arterial pressure, the optimal number of clusters should be 2, when the average silhouette coefficient is the highest. However, the within clusters sum of squares are too large when the number of clusters are equal to 2. As the number of clusters increases, the value of silhouette coefficient will be much lower. Hence, we chose 3 as the optimal number of clusters in systolic blood pressure and diastolic blood pressure clustering, 4 as the optimal number of clusters in mean arterial pressure clustering.

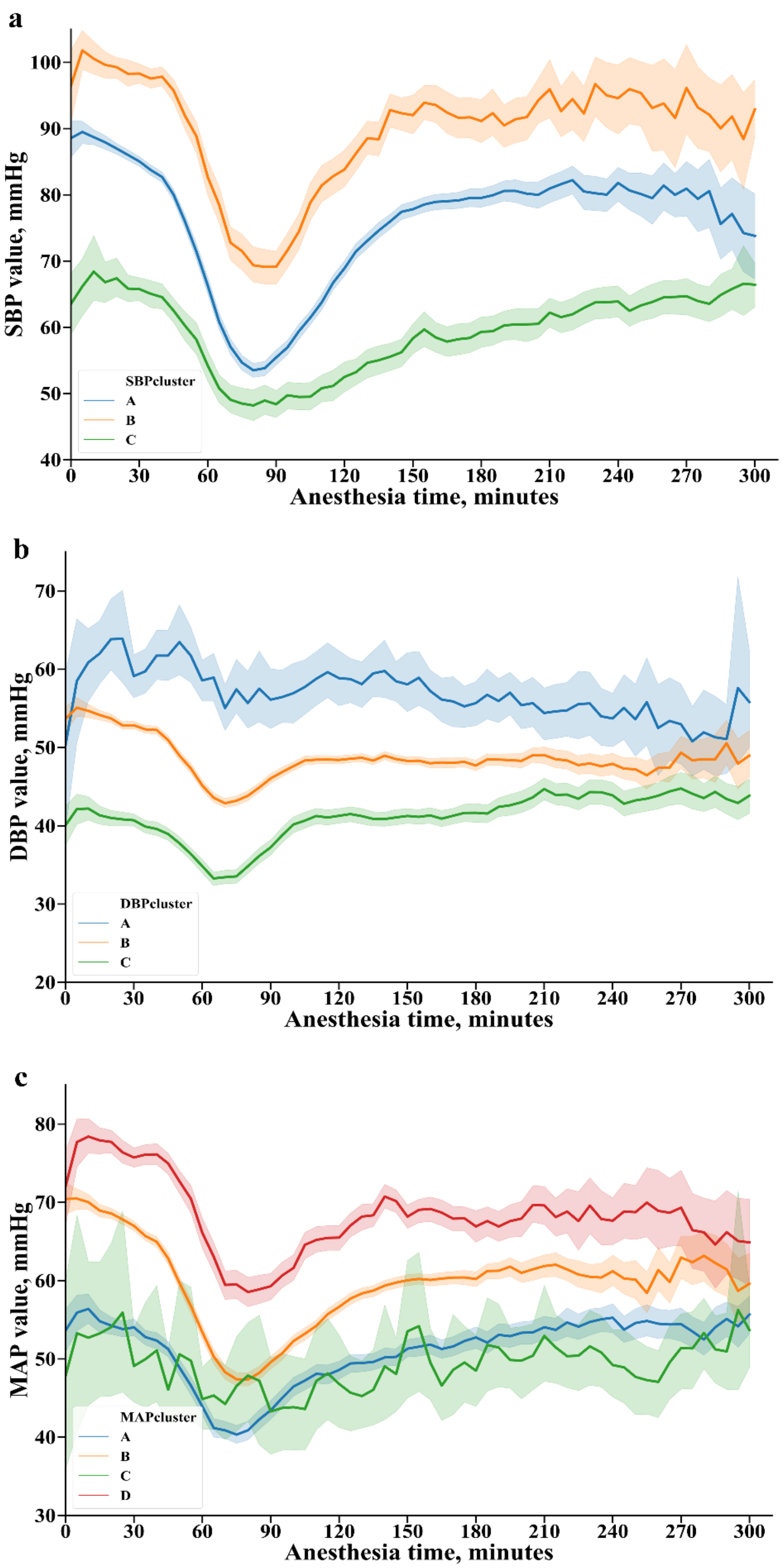

Supplementary Fig. S3. Mean and 95% confidence interval of blood pressure readings of different clusters. a, Systolic blood pressure; b, Diastolic blood pressure; c, Mean arterial pressure

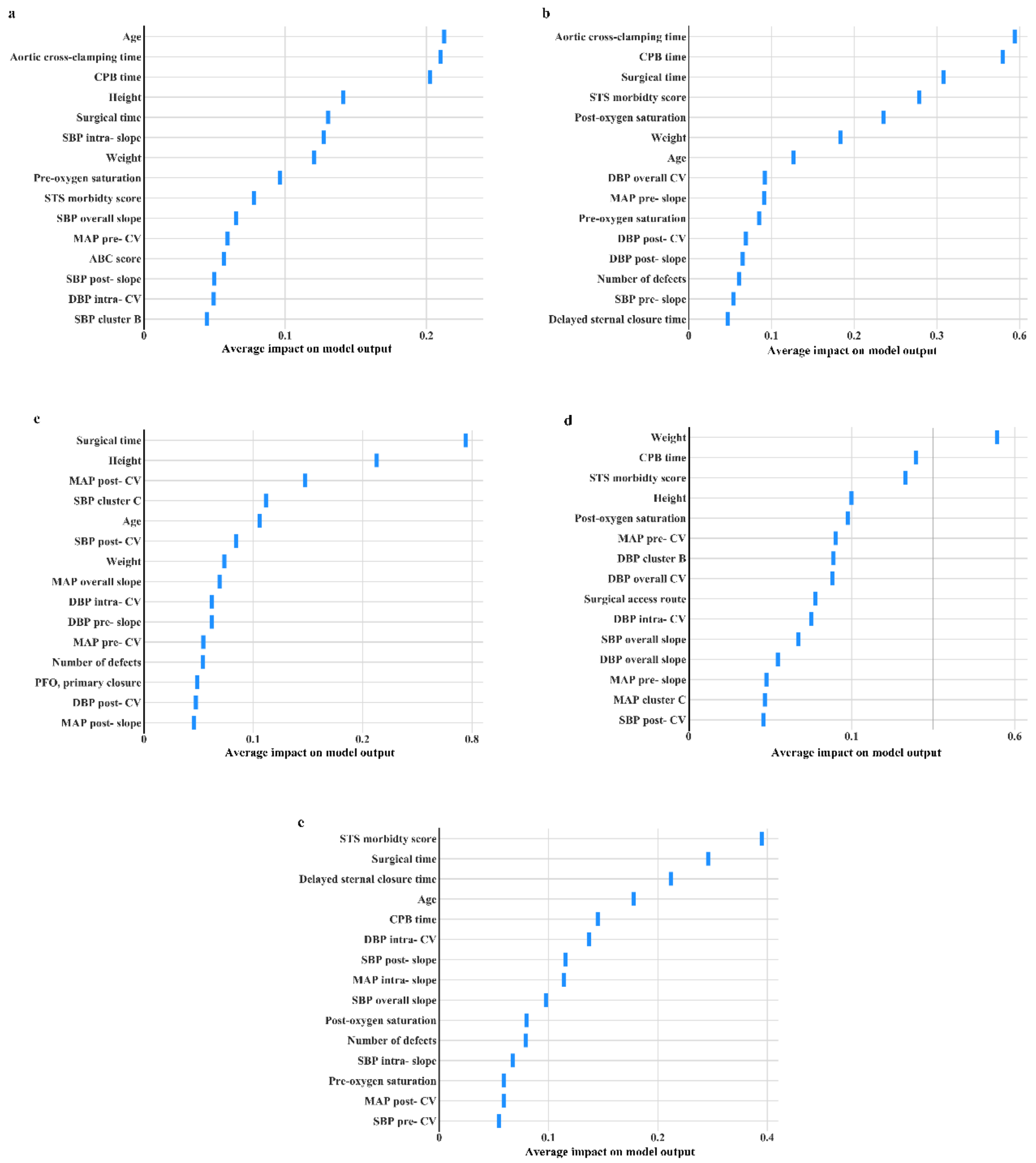

Supplementary Fig. S4. Averaged feature importance estimates by our proposed method for each category of complication. a, lung complication; b, cardiac complication; c, rhythm complication; d, infectious complication; f, other organ complication.

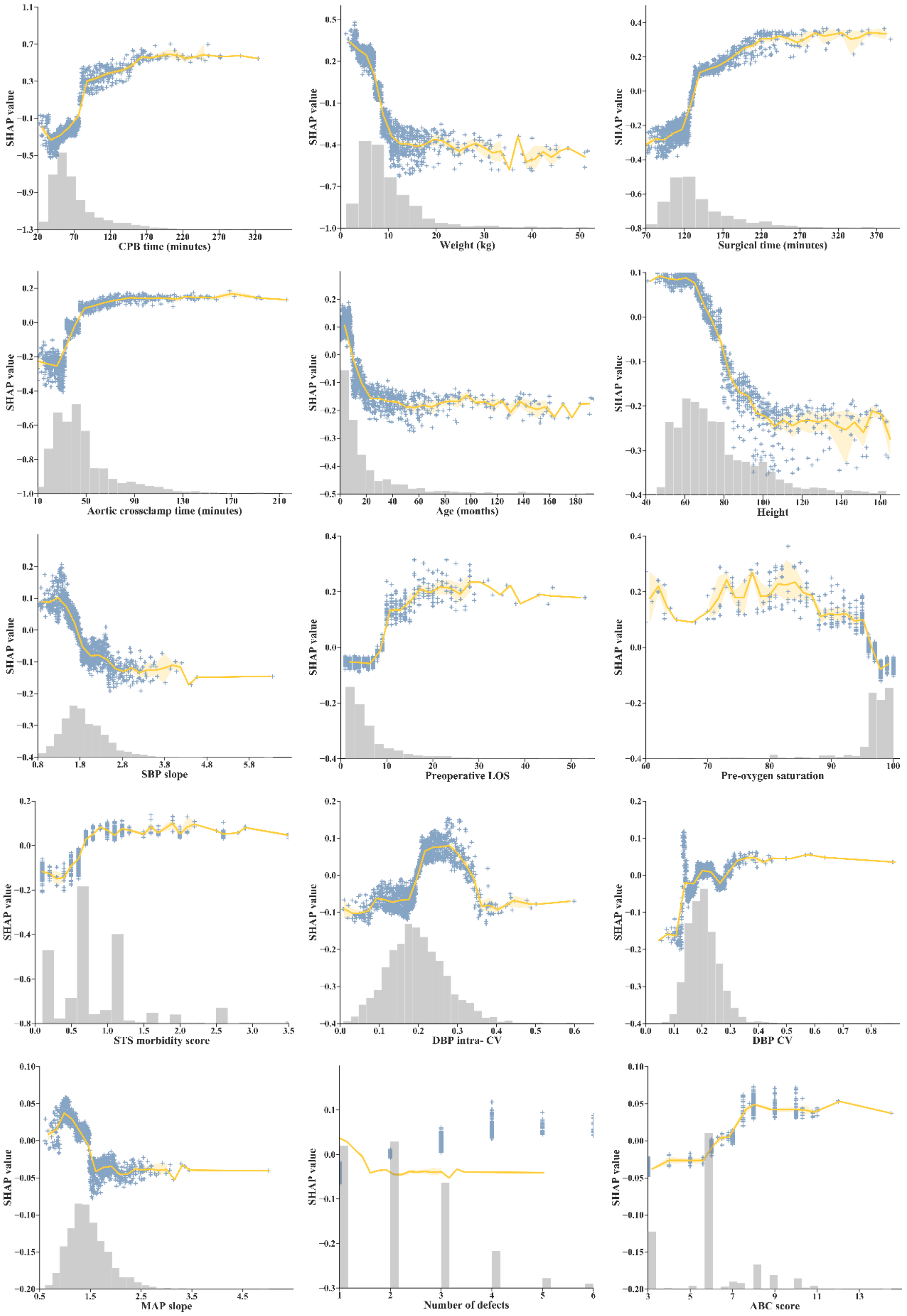

Supplementary Fig. S5. The influence of varying top 15 features values at different phases on complication prediction. These plots show the relationship between feature value and SHAP value. The grey histograms on each plot show the distribution of values for that feature in the training dataset. The orange line and shade represent the mean

and 95% confidence interval of the regression line
